# Breath‐holding treatments in tomotherapy with active breathing coordinator: A first case report

**DOI:** 10.1002/acm2.70270

**Published:** 2025-09-29

**Authors:** April Meikwan Chow, Ashley Chi Kin Cheng, Louis Kit Yee Lee

**Affiliations:** ^1^ Medical Physics Division Department of Medical Innovation & Technology CUHK Medical Centre Shatin Hong Kong SAR China; ^2^ CUHK Medical Centre Shatin Hong Kong SAR China; ^3^ Department of Clinical Oncology Faculty of Medicine CUHK Shatin Hong Kong SAR China; ^4^ Department of Medicine and Therapeutics Faculty of Medicine CUHK Shatin Hong Kong SAR China

**Keywords:** active breathing coordinator, breath‐holding treatment, tomotherapy

## Abstract

**Purpose:**

Respiratory motion poses a significant challenge in radiation therapy for thoracic and abdominal malignancies. For tomotherapy machines, it is even more challenging due to the 10‐s warm‐up time before initiating the treatment beams. A recent tomotherapy system upgrade has reduced this warm‐up time to 0.5 s for the TomoDirect delivery mode, enabling the feasibility of performing breath‐holding treatments. In this study, we investigated the feasibility of performing breath‐holding treatments on a tomotherapy machine with spirometry technique.

**Methods:**

A patient with gastric mucosa‐assisted lymphoid tissue lymphoma was treated with deep inspiration breath holding (30 Gy in 20 fractions) on a tomotherapy machine with Active Breathing Coordinator™. An in‐house visual system, featuring a compact 7″ LCD monitor, was implemented to provide visual feedback, allowing the patient to self‐monitor their breathings and adhere to the prescribed pattern.

**Results:**

The patient tolerated the breath‐hold well for the entire treatment. The number of breath‐holds for treatment was 9. The beam‐on time for each field ranged from 12.4 to 19.7 s, average 15.2 s.

**Conclusions:**

We have shown that BH treatment is feasible using an upgraded tomotherapy machine (Radixact v3.5) with Active Breathing Coordinator. Our proposed workflow includes an in‐house visual system that allows patients to visualize their breathing patterns on‐screen. This technique provides a practical solution for patients with thoracic and abdominal malignancies, addressing respiratory motion while minimizing radiation exposure for certain diseases.

## 1 INTRODUCTION

Respiratory motion has been a major concern for radiation therapy for patients with malignancies in the thorax and abdomen regions.^1^, ^2^ Motion‐induced dose uncertainties have been extensively studied and strategies such as 4‐dimensional (4D) treatment planning,^3^, ^4^ abdominal compression,^5^, ^6^ breath‐holding (BH),^7^, ^8^, ^9^, ^10^ gating,^11^, ^12^, ^13^, ^14^ tumor tracking,^15^, ^16^, ^17^, ^18^, ^19^ and combinations of these techniques have been proposed.

BH treatment utilizes approaches to manage respiratory motion during treatment delivery by temporarily inhibiting such motion. BH significantly reduces tumor motion and changes internal anatomy in a way that often protects critical normal tissues.^1^ For example, during inhalation the diaphragm pulls the heart posteriorly and inferiorly away from the breast, and thus potentially reduces the doses to the heart and lung.^20^, ^21^ Therefore, deep inspiration breath holding (DIBH) is becoming the standard radiotherapy treatment regime in patients with left‐sided breast cancer.^22^

Several approaches are available for BH, including spirometry,^23^, ^24^ surface guidance,^25^, ^26^ X‐ray guidance,^27^, ^28^ remote chest wall monitor,^29^, ^30^ and physical chest wall monitor.^31^, ^32^, ^33^ According to a recent survey by AAPM,^22^ the most frequently used device is the surface guidance system, followed by the remote chest wall monitor and spirometry.

The spirometry technique employs spirometer to measure the patient's lung volume and therefore monitor the breathing cycles during radiotherapy treatment.^34^ When connecting with conventional linear accelerator, it can also control the treatment beam automatically according to predefined threshold values. Commercial products include Active Breathing Coordinator™ (ABC) (Elekta, Stockholm, Sweden) and SDX® (DYN'R Medical Systems, Muret, France).

Traditional tomotherapy machines were programmed with a 10‐s warm‐up time before each treatment beam initiation,^23^ limiting the practicability of implementation of BH delivery. With the recent system upgrade, the 10‐s warm‐up time has been reduced to 0.5 s for TomoDirect delivery mode, thus enabling the feasibility of performing BH treatments.

In this study, we investigate the feasibility of performing BH treatments at tomotherapy machine with spirometry technique to gate the treatment beam manually. Using this technique, we describe the treatment and dosimetric parameters in one patient treated under DIBH.

## 2 METHODS

A patient (male; age = 60) diagnosed with gastric MALT (mucosa‐assisted lymphoid tissue) lymphoma treated with DIBH (30 Gy in 20 fractions) on a tomotherapy machine (Radixact X9; Accuray, Madison, Wisconsin, USA) was analyzed. This retrospective study was approved by the institutional Clinical Research Ethics Committee.

### 2.1 Motion management technique

Respiratory motion management technique used was DIBH using ABC (Active Breathing Coordinator R3.0; Elekta, Stockholm, Sweden). The ABC system consists of a digital spirometer to measure the respiratory curve, which is in turn connected to a balloon valve to suspend breathing at any predetermined position.^1^ In‐house visual system was implemented using compact 7″ LCD monitor mounted on a 3D‐printed stand. The LCD monitor was configured to mirror the display of the ABC software to provide visual information enabling patients to self‐monitor their own breathings to comply with prescribed breathing pattern.

### 2.2 Breath‐hold training

The patient underwent breath‐hold training in the simulation room in treatment position. The patient was familiarized with the ABC equipment, and instructed to hold his breath under deep inspiration. The training involved a series of inhalation, DIBH and exhalation exercises for at least 20 min, comparable to the total treatment time. During the training, the radiation therapists would observe the patient respiratory curve in the ABC software for patient comfort and stability. The ABC patient settings, including breath‐hold duration and respiratory volume, were also recorded. The patient was then asked to do self‐training at home by performing a series of inhalation, DIBH and exhalation exercises for at least 20 min every day prior to simulation, and till the start of the radiotherapy treatment. Radiotherapy planning was initiated if the patient can hold his breath for more than 25 s.

### 2.3 Simulation

The patient was instructed to fast for 6 h prior to simulation. For simulation, the patient was immobilized on a vacuum cushion. Helical CT scans of 3‐mm slice thickness were acquired using CT‐Simulator (SOMATOM Confidence, Siemens Healthineers, Erlangen, Germany) in free breathing and then in DIBH. For the latter, the respiratory volume under DIBH was recorded. The threshold volume for future treatment sessions was set as 0.8 of the recorded respiratory volume to achieve reproducible internal organ displacement while maintaining patient comfort.^35^

### 2.4 Treatment planning

The target volumes and organs‐at‐risk were delineated in DIBH scans by experienced oncologist and dosimetrists respectively. The clinical target volume to planned target volume margin used was 3 mm. The organs‐at‐risk were contoured per Radiation Therapy Oncology Group guidelines. Subsequently, treatment plan was generated using the Precision treatment planning system with VOLO™ Ultra planning and optimization algorithm (Accuray, Madison, Wisconsin, USA), fulfilling prescription parameters. The DIBH plan was generated using a 2.5 cm jaw opening with 9 fields under TomoDirect delivery mode. The automatic pitch calculation was used to determine the helical pitch for minimal thread effect. With the VOLO™ Ultra algorithm, the beam‐on time can be manipulated by adjusting parameters such as “Accelerated Treatment” or “Max. Beam On Time” during plan optimization.^36^ In this study, efforts were made to limit the beam‐on time for each field to be within the patient's BH capability by adjusting the “Max. Beam On Time”. Quality assurance checks of the treatment plan were performed prior to treatment as per institutional protocol.

### 2.5 Image guidance protocol and treatment delivery

The patient was instructed to fast for 6 h prior to every treatment fractions. For treatment delivery, the patient was positioned on the treatment couch in the treatment position, and a kilovoltage computed tomography (kVCT) was acquired under DIBH for position verification for every fractions. The imaging parameters were Anatomy = Thorax, Mode = Normal, FOV = 270 mm, Body Size = Medium and Scan Length = 169 mm. The scan duration was 20.0 s, which was set as per the patient's BH capability. The replicability of DIBH was confirmed using the ABC with the recorded threshold volume determined during breath‐hold training and simulation.

The patient was instructed to hold the breath under DIBH during beam‐on. Tomotherapy pause and resume function was utilized to control beam‐on and beam‐off manually according to the display of the ABC software which showed the respiratory volume of the patient.

## 3 RESULTS

**FIGURE 1:**
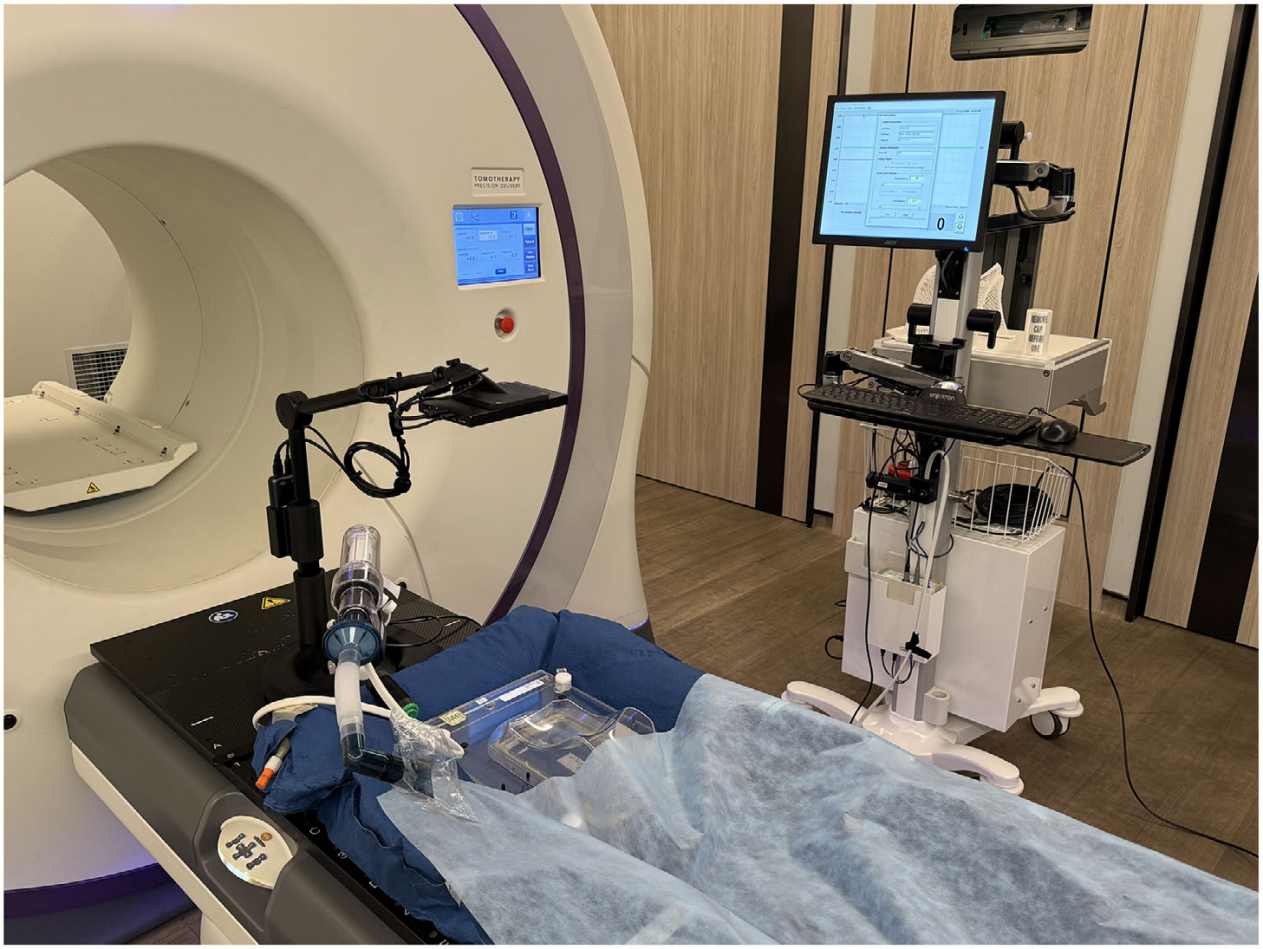
Photo showing the ABC setup with the in‐house visual system.

We treated one patient diagnosed with gastric MALT lymphoma using this technique under DIBH.^9^, ^37^ The patient tolerated well for the entire treatment. The ABC setup with in‐house visual system is illustrated in Figure 1.

### 3.1 Dosimetric parameters

**FIGURE 2:**
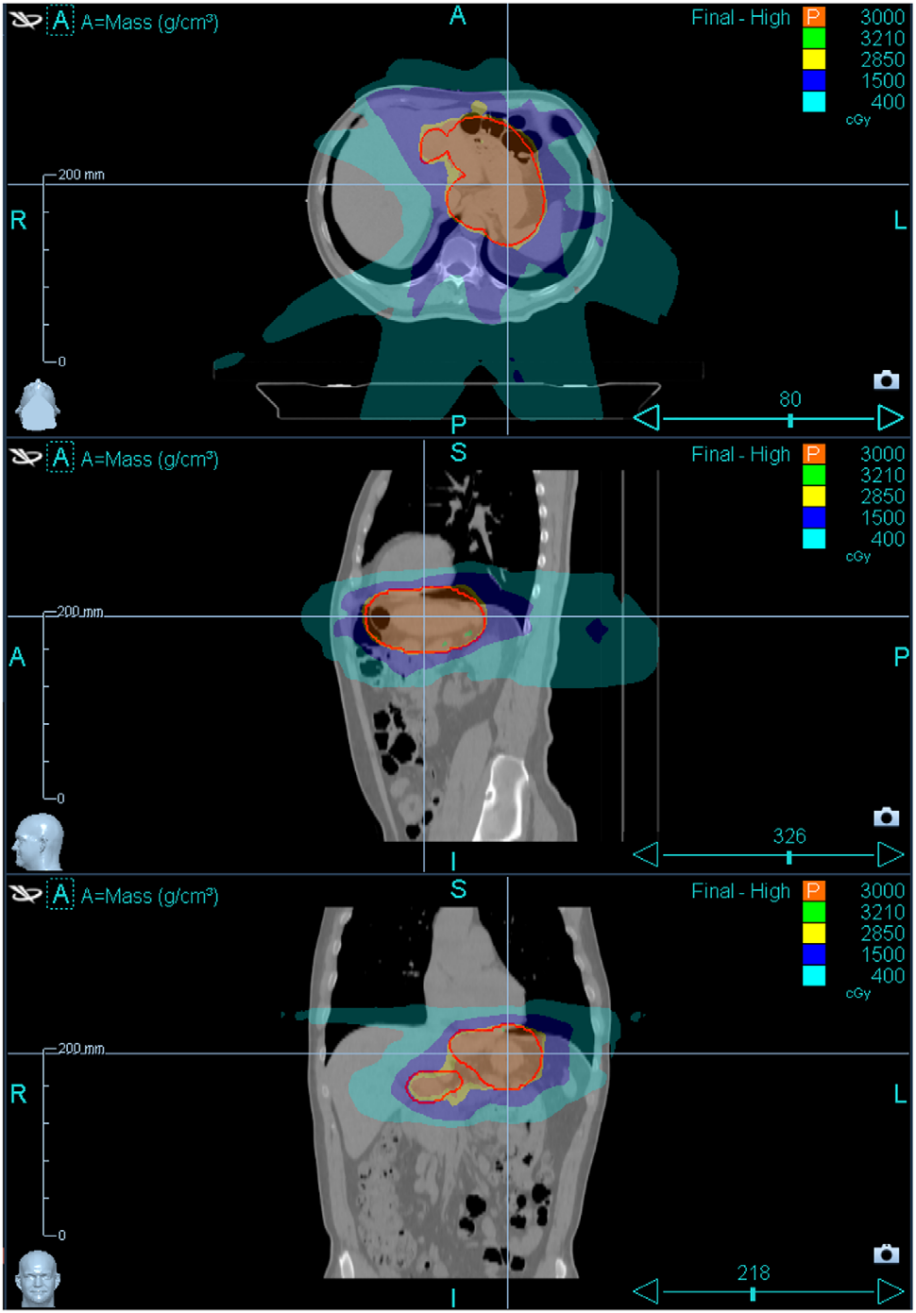
Axial, sagittal and coronal planes for the dose distribution of the treatment plan.

The target‐volume prescription goals and OAR constraints are met and shown in Table 1. The representative dose planes are displayed in Figure 2.

**TABLE 1:** Dosimetric criterion of the target and OARs.

Structure	Dosimetric criterion	Value
PTV	V_30Gy_ > 95%	95.6%
	V_27.9 Gy_ > 99%	99.7%
	D_max_ < 115%	109.1%
Spinal cord	D_max_ ≤ 39.08 Gy	22.3 Gy
Spinal cord_PRV	D_max_ < 43.24 Gy	24.79 Gy
Esophagus	D_mean_ < 30.92 Gy	11.21 Gy
Lungs_partial	V_18.65 Gy_ ≤ 30%	4.3%
	D_mean_ ≤ 18.65 Gy	3.88 Gy
Heart	V_23.07 Gy_ < 10%	6.7%
	D_mean_ < 23.95 Gy	1.65 Gy
	D_33%_ < 53.07 Gy	2.90 Gy
	D_67%_ < 40.36 Gy	1.16 Gy
	D_100%_ < 36.08 Gy	0.5 Gy
Liver	D_mean_ < 25.7 Gy	10.52 Gy
Kidney_L	D_mean_ < 14.16 Gy	4.79 Gy
Kidney_R	D_mean_ < 14.16 Gy	2.86 Gy
Stomach	D_max_ < 48 Gy	32.17 Gy
	D_2%_ < 44.61 Gy	31.83 Gy
	D_25%_ < 40.36 Gy	31.32 Gy
Duodenum	D_0.1cc_ < 58.88 Gy	32.16 Gy
	D_10cc_ < 40 Gy	31.19 Gy
Bowel space	V_40.36 Gy_ < 195 cc	0 cc

### 3.2 Treatment parameters

**FIGURE 3:**
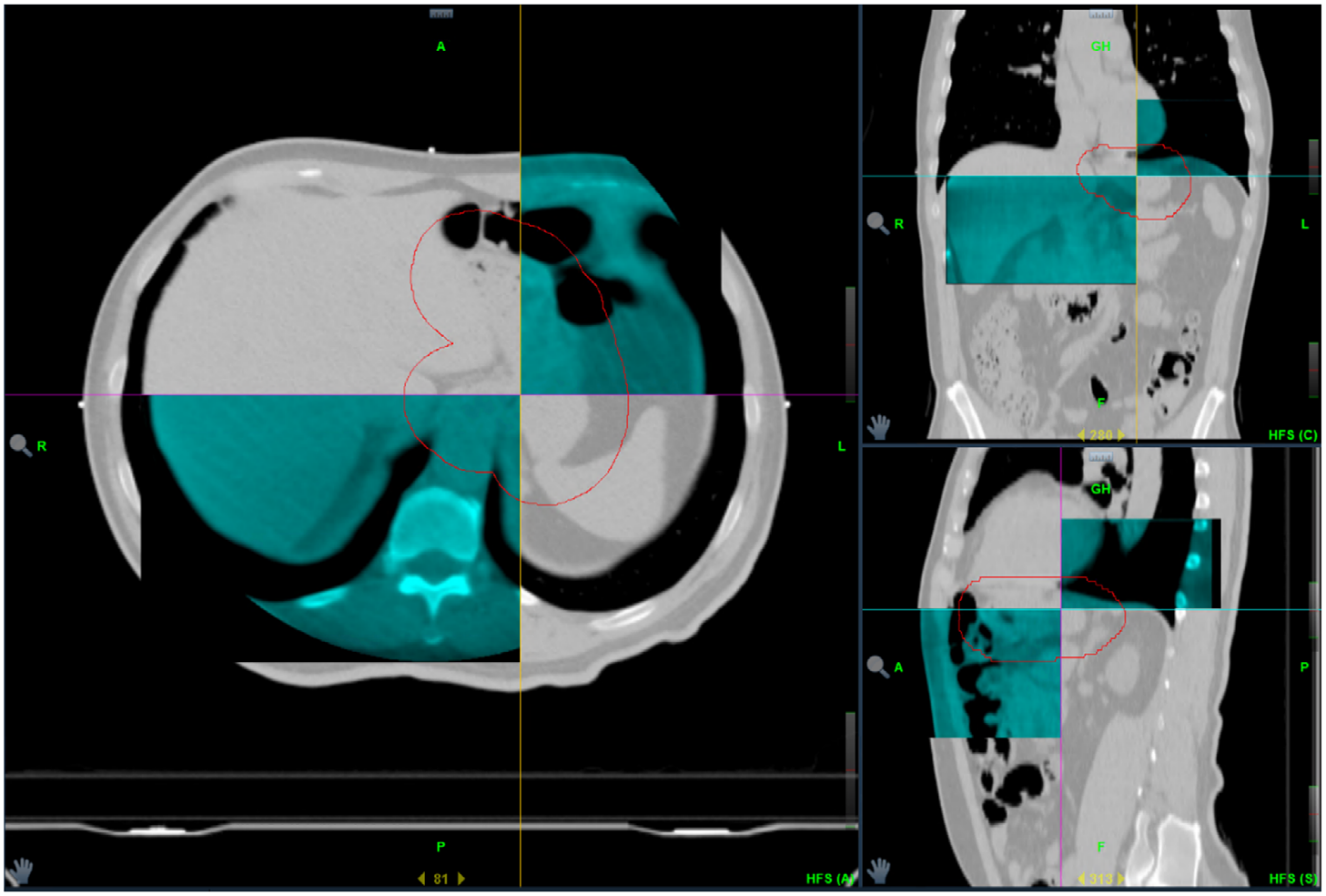
Axial, sagittal and coronal planes of a set of typical matching images for positional verification. grey color: planning CT images; cyan colour: kVCT images taken during image guidance.

The kVCT images provided information about the internal anatomy under DIBH for daily patient position verification. A set of typical matching images was illustrated in Figure 3.

The number of breath‐holds for treatment was 9, that is, the radiation therapists have to pause the treatment for 8 times in between fields. The beam‐on time for each field ranged from 12.4 to 19.7 s, average 15.2 s. Details are summarized in Table 2. The average total treatment time from patient entering the treatment room to completion of treatment was 24 min (range: 9–43 min).

**TABLE 2:** Summary of the plan delivery.

Field	Gantry angle	Beam on time (s)
1	0°	16.3
2	40°	12.6
3	80°	14.0
4	120°	17.6
5	160°	16.1
6	200°	14.5
7	240°	12.4
8	280°	13.3
9	320°	19.7

## 4 DISCUSSION

We presented a workflow to deliver BH treatment on a tomotherapy machine. To our understanding, this is the first study reporting the use of ABC on a tomotherapy machine.

BH treatment has become a standard motion management solution for patients with malignancies in the thorax and abdomen regions. However, most of the studies involved conventional linear accelerator coupled with various devices. Recently, tomotherapy launched a system upgrade to integrate a commercial SGRT system on the tomotherapy machine for automatic BH treatment deliveries.^38^, ^39^ With this system upgrade, the traditional 10‐s warm‐up time has been reduced to 0.5 s for TomoDirect delivery mode, enabling the feasibility of performing BH treatments. Before the SGRT package is available at our center, we developed a workflow to combine the use of ABC on the tomotherapy machine for BH treatments. We also developed an in‐house visual system for patients to view the on‐screen breathing pattern of the ABC system in a real‐time manner for the use of ABC with tomotherapy machine for BH treatments. With adequate coaching, the patient can learn how to understand the information on the screen (including whether his/her breathing is deep enough and how much time is left for the current BH) and how to co‐operate with the operators for the BH treatments. However, the ABC system only couples to selective linear accelerators for automatic beam on/off. When using with tomotherapy machine, user is required to control beam‐on and beam‐off manually. The planning session and subsequent treatment sessions require careful operation of the radiation therapists according to the previous information obtained about the patient's BH capability. Moreover, if the patient cannot tolerate the BH status towards the end of the BH period, the reaction of the operator to turn off the treatment beam may be delayed and unnecessary exposures may be delivered. This manual beam‐on and beam‐off steps may also introduce stress to the operators. It would be advantageous if the BH system can be coupled with the tomotherapy machine to control the beam automatically.

In this study, a 2.5 cm jaw opening was used for this particular plan to balance between plan quality and beam‐on time. Use of 5.0 cm field width may further reduce the beam‐on time, this may be more beneficial in treatment plans of stereotactic body radiation therapy which requires a higher dose per fraction.

The upgraded tomotherapy system only shortened the beam‐on warm‐up time in TomoDirect deleivery mode, the preparation time for TomoHelical delivery and kVCT acquisition is still significant. Therefore, we limited our treatment to TomoDirect delivery to overcome this limitation. Previous studies on esophageal carcinoma showed that fixed gantry multiple beams TomoDirect plans were comparable to TomoHelical plans in terms of dosimetry.^40^ As for kVCT, the imaging would not start immediately, but would only start after 4–5 s after pressing the beam‐on button. During BH training, the radiation therapists would gain knowledge about the time required for the patient to reach DIBH position after receiving instruction. Efforts were made to coincide the initiation of DIBH and the start of kVCT imaging to ensure that the kVCT was performed at DIBH position. Since it is unable to perform kVCT imaging in multiple BHs with tomotherapy machine, the kVCT scan duration would therefore be limited to one single BH duration. Imaging parameters should be chosen such that the treatment region could be scanned within one single BH duration. It would be beneficial if future upgrade of the system includes kVCT imaging capability across multiple BHs.

The ABC system only measures the volume inhaled and exhaled by the patient; it doesn't provide any information of the patient's anatomy during the BH. In our study, we did pre‐treatment verification kVCT to check the patient's anatomy against the planning CT. However, the anatomy of the patient during treatment is not being monitored. SGRT system may provide more information and confidence as it measures patient's surface continuously during BH.

Previously, Nangia et al. suggested to use a frame‐based tactile feedback system to gate DIBH treatment at tomotherapy manually^41^; however, the solution required in‐house fabrication of the feedback system. In our study, we demonstrated that BH treatments can be delivered with the use of ABC (a commercially available product) on tomotherapy machine. It offered a practical approach for the tomotherapy community to adopt in delivering BH treatments. The proposed workflow is suitable for patient patients with malignancies in the thorax and abdomen regions, for example, breast, lung, liver and pancreatic cancers.

## 5 CONCLUSION

In this study, we have demonstrated BH treatment is feasible on an upgraded tomotherapy machine (Radixact v3.5) with ABC. Our suggested workflow also includes an in‐house visual system for patients to view the on‐screen breathing pattern of the ABC system. This technique offers a practical solution for patients with malignancies in the thorax and abdomen regions to tackle the respiratory motion and minimizing radiation exposure for certain diseases.

## AUTHOR CONTRIBUTIONS

The authors confirm their contribution to the paper as follows: Study conception and design: April Meikwan Chow, Ashley Chi Kin Cheng, Louis Kit Yee Lee. Data collection: April Meikwan Chow, Ashley Chi Kin Cheng. Analysis and interpretation of results: April Meikwan Chow, Ashley Chi Kin Cheng, Louis Kit Yee Lee. Draft manuscript preparation: April Meikwan Chow, Louis Kit Yee Lee. All authors reviewed the results and approved the final version of the manuscript.

## CONFLICT OF INTEREST STATEMENT

The authors have nothing to report.
